# Interleukin-1β induces human cementoblasts to support osteoclastogenesis

**DOI:** 10.1038/ijos.2017.45

**Published:** 2017-12-13

**Authors:** Nam C-N Huynh, Vincent Everts, Prasit Pavasant, Ruchanee S Ampornaramveth

**Affiliations:** 1 Microbiology Department, Research Unit on Oral Microbiology and Immunology, Faculty of Dentistry, Chulalongkorn University, Bangkok, Thailand; 2 Mineralized Tissue Research Unit, Faculty of Dentistry, Chulalongkorn University, Bangkok, Thailand; 3 Department of Dental Basic Sciences, Faculty of Odonto-Stomatology, University of Medicine and Pharmacy at Ho Chi Minh City, Ho Chi Minh City, Vietnam; 4 Department of Oral Cell Biology, Academic Centre for Dentistry Amsterdam (ACTA), University of Amsterdam and VU University Amsterdam, Amsterdam, The Netherlands; 5 Department of Anatomy, Faculty of Dentistry, Chulalongkorn University, Bangkok, Thailand

**Keywords:** cementoblast, interleukin-1β, osteoclast, receptor activator of nuclear factor kappa-B ligand, tooth resorption

## Abstract

Injury of the periodontium followed by inflammatory response often leads to root resorption. Resorption is accomplished by osteoclasts and their generation may depend on an interaction with the cells in direct contact with the root, the cementoblasts. Our study aimed to investigate the role of human cementoblasts in the formation of osteoclasts and the effect of interleukin (IL)-1β hereupon. Extracted teeth from healthy volunteers were subjected to sequential digestion by type I collagenase and trypsin. The effect of enzymatic digestion on the presence of cells on the root surface was analyzed by histology. Gene expression of primary human cementoblasts (pHCB) was compared with a human cementoblast cell line (HCEM). The pHCBs were analyzed for their expression of IL-1 receptors as well as of receptor activator of nuclear factor kappa-B ligand (RANKL) and osteoprotegerin (OPG). In a co-culture system consisting of osteoclast precursors (blood monocytes) and pHCBs, the formation of osteoclasts and their resorptive activity was assessed by osteo-assay and ivory slices. The cells obtained after a 120 min enzyme digestion expressed the highest level of bone sialoprotein, similar to that of HCEM. This fraction of isolated cells also shared a similar expression pattern of IL-1 receptors (IL1-R1 and IL1-R2). Treatment with IL-1β potently upregulated RANKL expression but not of OPG. pHCBs were shown to induce the formation of functional osteoclasts. This capacity was significantly stimulated by pretreating the pHCBs with IL-1β prior to their co-culture with human blood monocytes. Our study demonstrated that cementoblasts have the capacity to induce osteoclastogenesis, a capacity strongly promoted by IL-1β. These results may explain why osteoclasts can be formed next to the root of teeth.

## Tooth injury: Getting to the roots of injury-related degradation

An investigation into the interaction between tooth root cells and an inflammatory protein sheds light on root degradation following injury. Osteoclast cells digest old bone to release nutrients and recycle bone tissues in a vital process called bone resorption. Cementum, the mineral substance covering tooth roots, is not usually resorbed, but injury to the tissues surrounding roots often triggers inflammation followed by root degradation. To understand this phenomenon better, Ruchanee Salingcarnboriboon Ampornaramveth at Chulalongkorn University in Bangkok, Thailand, and co-workers investigated whether cementum cells can promote the formation of osteoclasts. They found that when cementum cells were treated with interleukin 1 beta, an inflammatory protein expressed at high levels in tissues following injury, levels of another protein needed for osteoclast formation increased. This boosted osteoclast formation around roots, resulting in root resorption

## Introduction

Bone and cementum share some similar characteristics such as structure and composition and also the cells responsible for their formation, osteoblasts and cementoblasts, are quite alike. In fact, cementoblasts express genes similar to those expressed by osteoblasts such as type I collagen, bone sialoprotein (BSP), osteopontin, dentin matrix acidic phosphoprotein 1 (DMP-1), osteocalcin and bone gla protein.^1^ Yet, there is at least one unique difference between the two mineralized tissues: bone is remodeled, whereas cementum is not. Under physiological conditions, both cellular and acellular cementum increase slowly in thickness, but the tissue is not digested similar to bone. In contrast to bone, the root surface does not harbor specialized cells capable of resorbing the cementum. However, as an effect of tissue injury induced by trauma, root resorption can occur.

Injury to periapical tissue can cause damage to periodontal cells (cementoblasts and/or periodontal ligament (PDL) cells) after which local formation of cementoclasts/osteoclasts may occur.^2^ During this process of injury, inflammatory cytokines are likely to be released at these sites. Interleukin (IL)-1β is one of the most prominent inflammatory cytokines, and it has been reported to have an important role in inflammatory bone loss of the periodontium. Its expression is elevated in gingival crevicular fluid at sites of recent bone and attachment loss in patients with periodontal disease.^3^ An incubation of PDL fibroblasts with IL-1β resulted in an upregulation of receptor activator of nuclear factor kappa-B ligand (RANKL) expression.^4^ RANKL is essential for the formation of osteoclasts, and several studies have shown that these cells are indeed generated in the presence of PDL fibroblasts.^5-6^ It is not clear, however, whether cementoblasts also have the capacity to induce the formation of osteoclasts. Although osteoclast formation was shown to occur in a co-culture of osteoclast precursors and a mouse cementoblast cell line,^7^ no data are available on such a role played by primary human cementoblasts. In the present study, we investigated this by isolating cementoblasts from human third molars and their co-culture with mononuclear blood cells. Osteoclast formation as well as their resorptive activity was analyzed both in the absence and presence of IL-1β.

## Materials and methods

### Isolation and culturing of primary human cementoblasts

Third molars from healthy young individuals, aged 18–25 years, were extracted as recommended by their dentists. Each subject was without systemic and oral infection or diseases and the molars had no caries. The patients provided written consent for the use of discarded tissue for research purposes. Tissue samples were de-identified and analyzed anonymously. The Ethics Committee of the Faculty of Dentistry, Chulalongkorn University, Bangkok, Thailand approved the study to be carried out according to the protocol and informed permission dated and/or amended as follows in compliance with the ICH/GCP (HREC-DCU 2014-002). Immediately after extraction, each tooth was transferred to the laboratory in ice-cold storage medium (10% fetal bovine serum (FBS), 1% L-Glutamine, 0.5 mg⋅mL^−1^ gentamicin and 3 mg⋅mL^−1^ amphotericin B in Dulbecco’s modified Eagle’s medium (DMEM), #11960, Gibco, Life Technologies Corporation, Grand Island, NY, USA). The extracted molars were rinsed twice in Dulbecco’s phosphate-buffered saline without calcium and magnesium. The entire root surface was immersed in a digestion solution containing type I collagenase (2 mg⋅mL^−1^, #17100-017, Gibco, Life Technologies Corporation, Grand Island, NY, USA) and 0.25% trypsin in serum-free medium (1% bovine serum albumin in DMEM) and incubated at 37 °C. The released cells were collected every 30 min. The sequential digestion continued up to 150 min, thus included five consecutive digestion steps. The cells from each fraction were centrifuged and cultured in growth medium (10% FBS, 1% antibiotics in α-Minimum Essential Medium (αMEM), #12000-022, Gibco, Life Technologies Corporation, Grand Island, NY, USA). Primary human cementoblasts (pHCB) at the third to sixth passages were used for the experiments. hPDL cells were cultured from the first fraction of digestion (30 min). HCEM was a gift from Professor Takashi Takata (Hiroshima University). The cell line was cultured under the same conditions as used with the primary cells.

### Demineralized tooth section preparation

To examine the presence of cells that remained on the root surface following sequential enzymatic digestion, demineralized tooth sections of each time points were made and stained with hematoylin and eosin.

### IL-1β incubation

pHCBs and HCEM were seeded in 24-well plates. After 80% confluence, 1 or 10 ng⋅mL^−1^ recombinant human IL-1β (#201-LB-005, R&D Systems, Minneapolis, MN, USA) was added, and the cells were subsequently cultured for 24 h at 37 °C with 5% CO_2_.

### RNA isolation and semiquantitative reverse transcription-polymerase chain reaction (RT-PCR)

To assess the expression of marker genes, semiquantitative RT-PCR was performed as previously described.^8−9^ PCR primers for alkaline phosphatase (ALP), BSP and DMP-1 were used as markers of cementoblasts, IL-1β receptor types I and II (IL1-R1, IL1-R2) were used to screen endogenous expression in HCEM, pHCB and human periodontal ligament cells (hPDL cells). RANKL and osteoprotegerin (OPG) gene expression were used to evaluate the effect of IL-1β incubation (Table 1). All bands were scanned, analyzed and normalized with the expression of the housekeeping gene glyceraldehyde 3-phosphate dehydrogenase using the Bio-1D software version 15.03 (Vilber Lourmat, Marne La Vallée, France). Three independent experiments were repeated for each sample.

**Table 1:** Primers used in RT-PCR

Primer	Sequence ID	Sequence (forward and reverse 5′→3′)	Base pairs	Cycles
ALP	NM_000478.4	F: CGAGATACAAGCACTCCCACTTC R: CTGTTCAGCTCGTACTGCATGTC	121	35
BSP	NM_004967.3	F: GATGAAGACTCTGAGGCTGAGA R: TTGACGCCCGTGTATTCGTA	514	38
DMP-1	NM_004407.3	F: CAGGAGCACAGGAAAAGGAG R: CTGGTGGTATCTTGGGCACT	213	38
GAPDH	NM 002046.3	F: TGAAGGTCGGAGTCAACGGAT R: TCACACCCATGACGAACATGG	396	22
IL-1β	NM 000576.2	F: GGAGCAACAAGTGGTGTTCT R: AAAGTCCAGGCTATAGCCGT	458	32
IL1-R1	NM 000877.2	F: AGGAGACGGAGGACTTGTGT R: GCGTCATAGGTCTTTCCATC	755	30
IL1-R2	NM 173343.1	F: TCCTGCCGTTCATCTCATACC R: TCCATGTGCAAATCCTCTCTT	573	40
OPG	NM 002546.3	F: TCAAGCAGGAGTGCAATCG R: AGAATGCCTCCTCACACAGG	341	24
RANKL	NM 003839.2	F: GCCAGCTAGAAAACCACCAA R: TGGATTTGCTTCCAGGCTCA	517	40

ALP, alkaline phosphatase; BSP, bone sialoprotein; DMP-1, dentin matrix acidic phosphoprotein 1; GAPDH, glyceraldehyde 3-phosphate dehydrogenase; IL-1β, interleukin-1β; IL1-R1, interleukin-1 receptor 1; OPG, osteoprotegerin; RANKL, receptor activator of nuclear factor kappa-B ligand; RT-PCR, reverse transcriptase-polymerase chain reaction.

### Harvest of human peripheral blood monocytes (hPBMCs) and co-culture with cementoblasts

hPBMCs were isolated according to a previously described protocol.^4^ For co-culture experiments, human cementoblasts were seeded at 2.6 × 10^3^ cells⋅cm^−2^ in 24-well plates overnight. On the next day, the cells were incubated with 1 ng⋅mL^−1^ IL-1β for 24 h. On the following day, hPBMCs were seeded at 5 × 10^4^ cells⋅cm^−2^ onto the IL-1β-stimulated cementoblasts. Control cultures were cultured without the cytokine. The cultures were kept in αMEM, 10% heat-inactivated FBS (#16000-036, Gibco, Life Technologies Corporation, Grand Island, NY, USA), 1 × 10^−8^ mol⋅L^−1^,25-dihydroxyvitamin D3 (#D1530, Sigma-Aldrich, St Louis, MO, USA), 1 × 10^−7^ mol⋅L^−1^ dexamethasone and 25 ng⋅mL^−1^ recombinant human macrophage colony-stimulating factor (M-CSF; #574802, BioLegend, San Diego, CA, USA) for 21 days. A positive control group consisted of a monoculture of hPBMCs in the same medium plus 25 ng⋅mL^−1^ recombinant human RANKL (#591102, BioLegend, San Diego, CA, USA). A negative control group consisted of a monoculture of hPBMCs in αMEM supplemented with 10% FBS.

### Tartrate-resistant acid phosphatase (TRAcP) staining for osteoclasts

After 21 days of culture, the cells were fixed with 10% formalin for 5 min at room temperature and stained for TRAcP activity as well as for their nuclei (#MK300, TAKARA BIO, Shiga, Japan). Briefly, the cells were washed three times with distilled water, stained with chromogenic substrate (NABP/FRVLB) and sodium tartrate buffer and incubated at 37 °C for 45 min, followed by nuclear staining by methyl green for 5 min. Finally, the cells were washed with distilled water and analyzed for the presence of multinucleated TRAcP-positive cells (brightly red stained). Micrographs were taken from five different areas per well randomly. The number of TRAcP-positive multinucleated cells (≥3 nuclei) was assessed.

### Bone resorption assay *in vitro*

The functionality of the osteoclasts was analyzed by using two different resorption assays: Osteo-assay Plates (#3987, Corning, Corning, NY, USA) and ivory slices. The Osteo-assay plates are coated with inorganic three-dimensional crystalline material (bone biomimetic synthetic surface). Ivory slices were cut with a low speed water-cooled diamond saw. The slices had a diameter of 7 mm and a thickness of 0.5 mm. Osteoclasts were generated from hPBMCs in monoulture and co-cultures with cementoblasts as described above. After 21 days of culture, the cells were lysed by 2.5% chlorinated soda solution for 5 min at room temperature and washed by distilled water. Ivory slices were stained with a solution of 1% toluidine blue (#89640, Sigma-Aldrich, St Louis, MO, USA) in 1% sodium borate (#S9640, Sigma-Aldrich, St Louis, MO, USA) for 4 min. Resorption pits were observed and quantified using photomicrographs from five randomly chosen fields by inverted phase-contrast microscopy for Osteo-assay plates and stereomicroscopy Discovery V8 (Zeiss, Oberkochen, Germany) for the ivory slices.

### Data analysis

Each experiment was repeated at least three times. We calculated the means and s.d. for each set of data. For statistical analyses, independent samples comparison *t*-test, one-way analysis of variance, and Dunnett’s T3 *post hoc* tests were used to compare between groups using SPSS v.21 (IBM, New York, NY, USA) with the level of significance at *P*<0.05.

## Results

### Isolation and characterization of primary human cementoblasts

**Figure 1:**
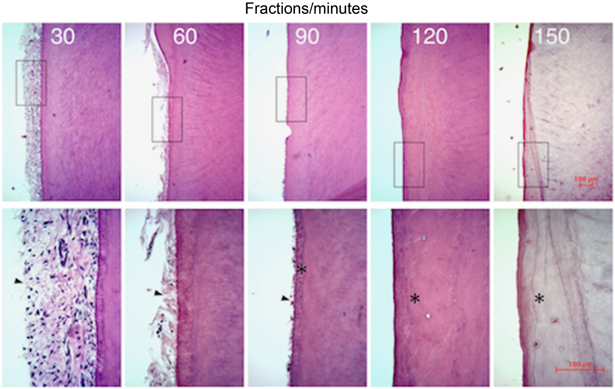
**Histological section, hematoxylin and eosin staining of the root surface after enzymatic digestion at each time point: 30, 60, 90, 120, and 150 min (arrowhead: cells, asterisk: cementum; scale bar 100 μm).**

In order to evaluate how enzymatic digestion affected the layer of cells on the root surface, histological sections of the teeth were examined. The thickness of ligament and cellular layer on the root surface gradually decreased with the increasing digestion time. At 90 min of digestion, only a single layer of cells remained associated with the cementum (Figure 1). After 150-min incubation, no cells was detected, indicating that the cells present at the 120-min time interval were the cementoblasts. These cells were isolated during the 120-min incubation.

**Figure 2:**
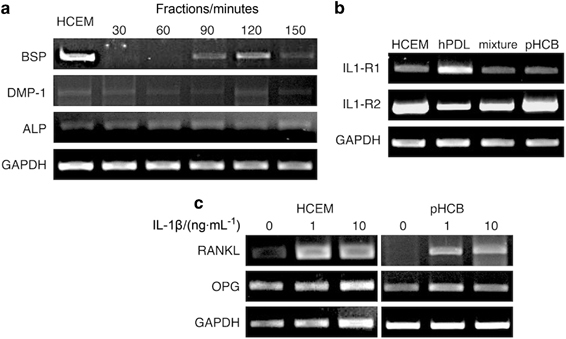
**mRNA expression of cells collected from different fractions of enzymatic digestion of tooth.** (**a**) BSP, DMP-1 and ALP expression. (**b**) IL-1 receptor 1 and receptor 2 expression. (**c**) RANKL and OPG expression after treatment with IL-1β (mixture: cells from 60–90 min fractions). ALP, alkaline phosphatase; BSP, bone sialoprotein; DMP-1, dentin matrix acidic phosphoprotein 1; GAPDH, glyceraldehyde 3-phosphate dehydrogenase; HCEM, human cementoblast cell line; hPDL, primary human periodontal ligament cells; IL, interleukin; OPG, osteoprotegerin; pHCB, primary human cementoblasts; RANKL, receptor activator of nuclear factor kappa-B ligand.

To characterize the cells from each fraction, they were compared with the HCEM, and the expression of BSP, DMP-1 and ALP was evaluated. BSP was not expressed in the early fractions (30 and 60 min). Low levels of expression were seen in the 90-min fraction and a peak was found in the 120-min fraction (Figure 2a). The expression of BSP of the latter cells was comparable to that of the HCEM. The expression of DMP-1 and ALP was comparable for all samples. The cementoblast phenotype corresponded to their BSP expression profile resembling that of the HCEM. We therefore considered the cells from 120-min fraction as primary human cementoblasts and used these for our further experiments.

### IL-1β treatment induced RANKL expression and increased the RANKL/OPG ratio in human cementoblasts

In order to investigate the response of cementoblasts to IL-1β, we examined first whether these cells expressed IL-1β and its receptors. pHCB, HCEM, hPDL and the cells from the 60- and 90-min isolations (those contained both hPDL and pHCB) did not express IL-1β (data not shown) but expressed both types of the IL1-R1 and IL1-R2. pHCB and HCEM expressed both IL1-R1 and IL1-R2, but the expression of IL1-R2 was stronger. This pattern was distinct from hPDLs; these cells expressed comparable levels of IL1-R1 and IL1-R2 (Figure 2b).

**Figure 3:**
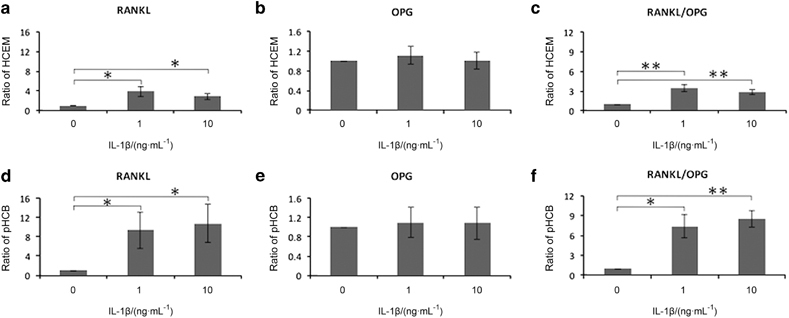
**The effect of IL-1β on osteoclast-associated genes.** (**a**) RANKL, (**b**) OPG and (**c**) RANKL/OPG ratio of HCEM. (**d**) RANKL, (**e**) OPG and (**f**) RANKL/OPG ratio of pHCB by semiquantitive RT-PCR (one-way ANOVA, Dunnett’s T3 *post hoc* test, *P<0.05; **P<0.01, *n*=4). ANOVA, analysis of variance; HCEM, human cementoblast cell line; IL, interleukin; OPG, osteoprotegerin; pHCB, primary human cementoblasts; RANKL, receptor activator of nuclear factor kappa-B ligand; RT-PCR, reverse transcriptase-polymerase chain reaction.

IL-1β increased RANKL mRNA expression in both pHCB and HCEM. There was no difference in OPG expression following IL-1β incubation (Figure 2c). These responses resulted in an increased ratio of RANKL/OPG (Figure 3).

### IL-1β stimulated cementoblast induced osteoclastogenesis

To determine whether cementoblasts had the capacity to induce osteoclastogenesis and the effect of IL-1β hereupon, they were co-cultured with osteoclast precursors, hPBMCs. Cementoblasts were or not precultured with IL-1β for 24 h prior to their co-culture with hPBMCs. The co-cultures were kept for 21 days in media containing M-CSF, Vit D3 and dexamethasone. Monocultures of hPBMCs were cultured in medium that contained RANKL, M-CSF, Vit D3 and dexamethasone. These latter cultures were used as positive control.

**Figure 4:**
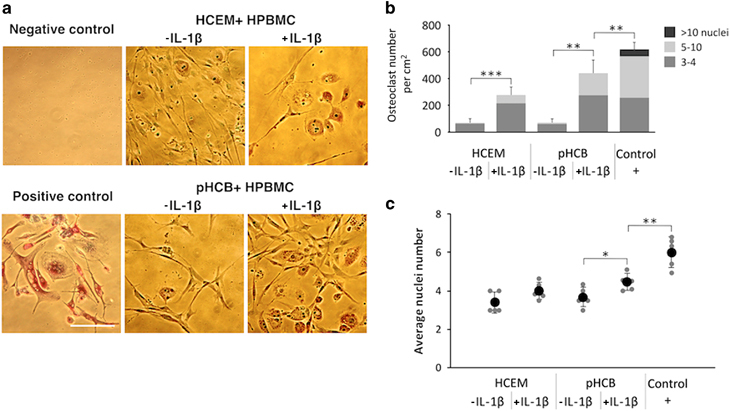
**Co-culture of cementoblasts and primary human peripheral blood monocytes for 21 days.** (**a**) TRAcP and nuclear staining, scale bar 100 μm. (**b**) Osteoclast number and the number of cells including different nuclei number. (**c**) The average number (black dots) of nuclei per generated osteoclasts (independent samples comparison *t*-test, *n*=5). HCEM, human cementoblast cell line; hPBMC, human peripheral blood monocyte; IL, interleukin; pHCB, primary human cementoblasts; TRAcP, tartrate-resistant acid phosphatase.

In the absence of IL-1β, TRAcP-positive multinucleated cells were present, but their numbers were low, being approximately 60 cellspercm^2^. A 24 h, pretreatment of the cementoblasts with IL-1β significantly increased the number of TRAcP-positive multinucleated cells (Figure 4a and 4b). Under these conditions, the number of TRAcP-positive cells with 3–4 and 5–10 nuclei was 275 and 170 cellspercm^2^, respectively. The osteoclasts generated in the co-culture groups were smaller than those generated in the presence of exogenous RANKL, the positive control group. The diameter of the osteoclasts generated in the presence of the cementoblasts was around 50 μm, whereas those generated in the presence of exogenous RANKL had a diameter of around 100 μm.

It was of interest to note that the osteoclasts generated in the co-culture with cementoblasts were found in close association with the latter cells (Figure 4a). The majority of osteoclasts generated in the co-cultures contained 3–4 nuclei; only a low percentage of cells had 5–10 nuclei. In the cultures of hPBMCs to which RANKL was added (positive control group), the majority of cells contained 5–10 nuclei or even >10 nuclei per cell. In summary, a pretreatment of pHCBs with IL-1β stimulated the formation of multinucleated osteoclasts. Yet, the addition of exogenous RANKL resulted in a higher number of TRAcP-positive cells with more nuclei per cell (Figure 4c).

### IL-1β pretreated cementoblasts induced functional resorbing osteoclasts

**Figure 5:**
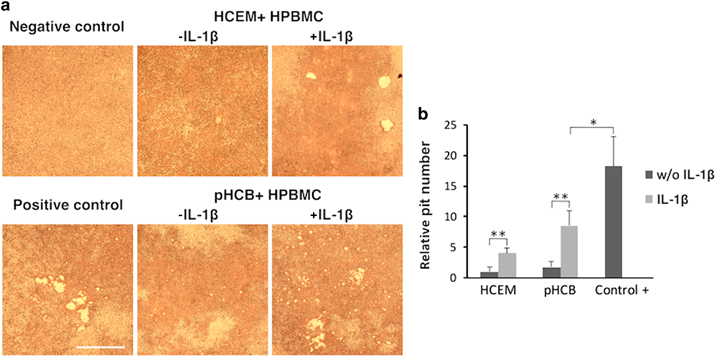
**Pit resorption assay by Osteo-Assay Plate: co-culture of cementoblasts and primary human peripheral blood monocytes for 21 days.** (**a**) Pit resorption (scale bar 100 μm). (**b**) Fold change of pit resorption number (independent samples comparison *t*-test, *n*=4). HCEM, human cementoblast cell line; hPBMC, human peripheral blood monocyte; IL, interleukin; pHCB, primary human cementoblasts.

**Figure 6:**
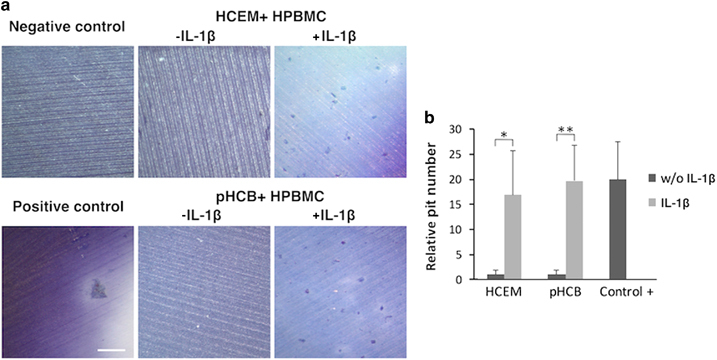
**Pit resorption assay by ivory slices: co-culture of cementoblasts and primary human peripheral blood monocytes for 21 days.** (**a**) Pit resorption stained with 1% toluidine blue in 1% sodium borate (scale bar 200 μm). (**b**) Fold change of pit resorption number (independent samples comparison *t*-test, *n*=4). HCEM, human cementoblast cell line; hPBMC, human peripheral blood monocyte; IL, interleukin; pHCB, primary human cementoblasts.

To analyze an important functional aspect of the multinucleated cells formed, resorption of a mineralized surface, two different types of pit assays were used. The cementoblasts were cultured as indicated above, but they were seeded on Osteo-assay plates or on ivory slices. Resorption was found in co-cultures with or without a preincubation with IL-1β (Figures 5 and 6). Yet, the co-cultures in which the cementoblasts were preincubated with the cytokine showed a significantly higher level of resorption (Figures 5 and 6). The positive control group (monoculture of hPBMCs in medium supplemented with RANKL) showed the highest level of resorption. Analysis of the ivory slices revealed the presence of resorption pits (Figure 6). The number of these pits was significantly higher in the IL-1β preincubated cementoblasts culture and comparable to the number in the positive control group (Figure 6).

## Discussion

Our study, for the first time, demonstrated the capacity of primary human cementoblasts to induce the formation of functional osteoclasts. We found that cementoblasts had the capacity to induce multinucleated TRAcP-positive cells that resorbed a mineralized substratum. Thus these cells were actual osteoclasts. An essential question is, however, whether the cells isolated from the tooth root surface were cementoblasts. By using a sequential enzymatic digestion, the cementum-lining cells were collected and characterized.^10-11^ In addition, histological analysis was performed to investigate the cells that remained on the root surface after each incubation. The results clearly demonstrated that in almost all samples after 120 min of digestion no cells occupied the root surface anymore. With some differences between specimens, we were able to obtain a very small number of cells from the 150-min fraction. This small amount proved to be enough for gene expression analysis but could not be used for further experiments. Therefore, cells collected in the 120-min fraction were considered as the last cellular layer lining the root surface, thus cementoblasts. The mRNA expression of BSP, a major protein component of cementum, confirmed the cementoblast phenotype. This expression was similar to that of an immortalized cell line used in a previous study.^12^ The cells from the outer layer, primarily PDL cells, did not express BSP, whereas the cells from 90-min fraction expressed a low level. These findings are in line with data presented previously on the expression of BSP by cementum-associated cells.^11-13^ The latter authors showed that BSP is primarily expressed by cells lining the root surface, the cementoblasts, but not by PDL cells. Our finding might indicate that cementoblasts resemble osteoblasts more than PDL cells.^8, 11^ Moreover, our pHCB expressed a similar pattern of DMP-1 and ALP mRNA as HCEM and PDL cells. Expression of DMP-1 has been shown for a cementoblast cell line and undifferentiated primary PDL cells.^8, 14-15^ Meanwhile, several studies showed that cementoblasts expressed a similar level of ALP mRNA as PDL cells but expressed a higher ALP activity.^11, 16-17^

To investigate whether the formation of osteoclasts as induced by cementoblasts was modulated by the pro-inflammatory cytokine, IL-1β, we first investigated whether human cementoblasts expressed IL-1β and its receptors. Apparently the cementoblasts and PDL cells did not express IL-1β. This finding is in line with other studies where only a weak expression of the cytokine by mesenchymal cells was noted, whereas high levels are produced by activated macrophages and various immune cells.^18^ Both IL-1β receptors were expressed at a comparable level by the cementoblasts and the cell line while the expression by hPDL cells differed. IL1-R1 generally mediates most of the responses to IL-1, whereas IL1-R2 has been reported to function as a decoy receptor.^19^ Our study demonstrated a difference in the expression of IL1-R1 and IL1-R2 in cementoblasts and PDL cells. The latter (decoy) receptor was higher expressed by cementoblasts. This finding might suggest that these two cell types respond differently to IL-1. PDL cells can stimulate osteoclastogenesis by expression of RANKL and inhibit this process by OPG. In this way, these fibroblasts can influence processes such as periodontitis and orthodontic tooth movement.^20^ The difference in expression of IL1-R1 and IL1-R2 might imply distinctive properties of cementoblasts and PDL cells. The higher expression of R2 receptor in cementoblasts might make the cells less sensitive to the inflammatory cytokine. Further studies are needed to explore this possibility in more detail.

In several studies using a mouse cementoblast cell line (OCCM-30), an upregulation of RANKL/OPG was shown to occur under the influence of different compounds, such as sclerostin and prostaglandin E_2_ (PGE_2_). These findings suggested that this cell type can indeed induce osteoclastogenesis.^7, 21-22^ Bao *et al.*^21^ demonstrated the inhibition of sclerostin on OPG expression in this mouse cementoblast cell line.^21^ Oka *et al.*^7^ showed that PGE_2_ promoted cementoblast-mediated cementoclastogenesis by regulating the expression of RANKL and OPG via the EP4 pathway.^7^ In contrast to our findings, Kim *et al.*^23^ reported that human cementoblasts do not have the capacity to induce osteoclastogenesis. These authors co-cultured, however, the cells with mouse bone marrow cells.^23^ A possible explanation for the lack of osteoclast formation is the source of osteoclast precursors used. It is not unlikely to assume that the interaction between human cementoblasts and mouse cells is quite different from an interaction of such cementoblasts with human blood-derived precursors, as used in our study.

Although macrophages and osteoclasts have been detected in the periodontal space, still little is known about the origin of newly recruited osteoclasts.^24^ Resorption requires specific interactions between various cell types and hard tissue. Multinucleated osteoclasts are formed as a result of injury to bone, cementum or dentin.^25^ Prior to the onset of osteoclastogenesis, osteoclast precursors are stimulated by M-CSF to undergo mitosis while RANKL/OPG modulates their differentiation into multinucleated osteoclasts.^26-27^ The ability of cementoblasts to induce osteoclast formation was enhanced if the cells were exposed to the inflammatory cytokine, IL-1β. The effect was seen already at fairly low concentrations of the cytokine. Previously it was shown that stimulation of PDL fibroblasts with 1, 10 and 100 ng⋅mL^−1^ IL-1β had a long-lasting effect, leading to a significantly increased osteoclastogenesis after a short and single exposure of these cells to the cytokine.^4^ Thus both types of periodontal cells, fibroblasts and cementoblasts, respond to the cytokine by inducing relatively high numbers of osteoclasts.

In our co-culture model, we found fewer and smaller osteoclasts when compared with a monoculture of osteoclast precursors with a continuous supply of recombinant RANKL. The number of nuclei per cell was also lower of osteoclasts generated in the co-cultures. This may represent osteoclasts with a somewhat lower resorptive activity. The difference in outcome is most likely due to the expression of RANKL induced by a single dose exposure to IL-1β. Such an expression is probably lower than the amount of added RANKL. It is questionable whether this approach mimics an actual pathological condition that occur *in vivo.* Under these conditions, the cells are probably continuously exposed to the inflammatory cytokine. Induction of osteoclasts in previous studies by co-culture with PDL cells, the average numbers of nuclei per osteoclast were higher than in our study, suggesting that PDL cells might have a stronger osteoclastogenic supporting ability compared with cementoblasts.^28^ The higher number in PDL co-cultures is in accordance with the stronger upregulation of RANKL/OPG in response to pro-inflammatory cytokines by PDL cells.^5-28^

Cementoblasts as well as the cementum itself appear to have an important role in protecting the root from resorption.^23^ Our study seems to support such a role of cementoblasts by demonstrating that without an inflammatory cytokine this cell hardly induced osteoclast formation. However, after exposure to IL-1β cementoblasts may switch their side to provide a stimulatory signal for osteoclastogenesis. This might be a crucial underlying mechanism of root resorption under pathological condition.

The multinucleated cells generated in the co-cultures proved to be functional osteoclasts. These cells not only dissolved the mineralized layer in the osteo-assay but also generated actual resorption pits in ivory slices. The formation of the pits is strong evidence for the nature of these cells being indeed osteoclasts. The demineralization of the osteo-assay plates could have been caused by macrophages,^29-30^ but the actual degradation of the ivory can only be performed by osteoclasts. The results showed further that IL-1β stimulated the resorption, an effect noted previously.^4^

The participation of cementoblasts in the formation of osteoclasts has been shown before. Diercke and co-workers^31-32^ showed an IL-1β-dependent induction of RANKL by human cementoblasts, but this was apparent only under compressive force mimicking orthodontic tooth movement.^31-32^ Nemeto *et al.*^33^ suggested that murine cementoblasts participate in the recruitment of osteoclastic precursors via Toll-like recceptor-2 under the influence lipopolysaccharide.

Direct cell–cell interaction between osteoclast precursors and PDL fibroblasts significantly modulates the cellular response, which favors the expression of osteoclast-related gene expression and the ultimate formation of osteoclasts.^4-34^ In fact, the presence of cementoblast- or fibroblast-like stromal cells in association with odonto/osteoclasts has been reported in root resorption of deciduous teeth.^35^ It is, however, not very likely that under physiological conditions cementoblasts and precursors of osteoclasts have a frequent chance to come into direct contact. Under pathological conditions, such a situation is more likely to occur.

Collectively, our study demonstrated the capacity of human cementoblasts to induce osteoclastogenesis, an effect strongly promoted by the pro-inflammatory cytokine IL-1β.
